# *Disrupted-in-Schizophrenia-1* is essential for normal hypothalamic-pituitary-interrenal (HPI) axis function

**DOI:** 10.1093/hmg/ddx076

**Published:** 2017-03-01

**Authors:** Helen Eachus, Charlotte Bright, Vincent T. Cunliffe, Marysia Placzek, Jonathan D. Wood, Penelope J. Watt

**Affiliations:** 1 Department of Animal and Plant Sciences, University of Sheffield, Western Bank, Sheffield S10 2TN, UK; 2 The Bateson Centre, Department of Biomedical Science, Firth Court, Western Bank, Sheffield S10 2TN, UK; 3 Sheffield Institute for Translational Neuroscience, Department of Neuroscience, University of Sheffield, Sheffield S10 2HQ, UK

## Abstract

Psychiatric disorders arise due to an interplay of genetic and environmental factors, including stress. Studies in rodents have shown that mutants for *Disrupted-In-Schizophrenia-1* (*DISC1*), a well-accepted genetic risk factor for mental illness, display abnormal behaviours in response to stress, but the mechanisms through which *DISC1* affects stress responses remain poorly understood. Using two lines of zebrafish homozygous mutant for *disc1*, we investigated behaviour and functioning of the hypothalamic-pituitary-interrenal (HPI) axis, the fish equivalent of the hypothalamic-pituitary-adrenal (HPA) axis. Here, we show that the role of DISC1 in stress responses is evolutionarily conserved and that DISC1 is essential for normal functioning of the HPI axis. Adult zebrafish homozygous mutant for *disc1* show aberrant behavioural responses to stress. Our studies reveal that in the embryo, *disc1* is expressed in neural progenitor cells of the hypothalamus, a conserved region of the vertebrate brain that centrally controls responses to environmental stressors. In *disc1* mutant embryos, proliferating *rx3+ *hypothalamic progenitors are not maintained normally and neuronal differentiation is compromised: *rx3*-derived *ff1b+ *neurons, implicated in anxiety-related behaviours, and *corticotrophin releasing hormone (crh)* neurons, key regulators of the stress axis, develop abnormally, and *rx3*-derived *pomc+ *neurons are disorganised. Abnormal hypothalamic development is associated with dysfunctional behavioural and neuroendocrine stress responses. In contrast to wild type siblings, *disc1* mutant larvae show altered *crh* levels, fail to upregulate cortisol levels when under stress and do not modulate shoal cohesion, indicative of abnormal social behaviour. These data indicate that *disc1* is essential for normal development of the hypothalamus and for the correct functioning of the HPA/HPI axis.

## Introduction

Phenotypes are shaped throughout the life-course by a complex interplay between genes and the environment. When homeostasis is threatened by environmental stress, animals respond adaptively by altering their metabolism, physiology and behaviour. These adaptive responses are co-ordinated by the hypothalamic-pituitary-adrenal (HPA) axis (1). Activation of the HPA axis promotes cortisol release and promotes adaptation (2,3). Circulating cortisol in turn triggers negative feedback systems that limit HPA axis function. However, this circuit can become reprogrammed to trigger responses that are seemingly maladaptive (4,5): in humans, HPA hyperactivity is linked to heightened risk for depression and anxiety disorders (6). Maladaptive stress responses can be triggered through wide-ranging insults, and increasing evidence suggests that insults in developmentally-sensitive periods predispose individuals to later heightened vulnerability to stress. For example, it is well documented that heightened stress in early life can result in the development of adult-onset psychiatric disorders in humans (1,4,7–10). At the same time, the stress response is modulated by an individual’s genetic makeup, and genotype is thought to contribute to individual differences in susceptibility to psychiatric disorders (11–14). However, whilst animal models have demonstrated that ablation of individual genetic components of the HPA axis can affect stress phenotypes and behaviour (15–18), no study has yet shown a direct link between genetic regulation of HPA axis development and maladaptive stress responses.

One well established genetic risk factor for human psychiatric illness, *Disrupted-In-Schizophrenia-1* (*DISC1)*, was originally identified at a chromosomal translocation breakpoint in a single Scottish family, in which a high proportion of family members suffered from mental illness (19). Some translocation carriers showed a range of clinical phenotypes, including schizophrenia, major depression and bipolar disorder, whilst other carriers had no psychiatric diagnosis (19). Individuals carrying this translocation, including those with no psychiatric condition, exhibited a defect in their cognitive function during decision-making processes (P300 event-related potential), a trait considered to be a marker for risk for schizophrenia (20). The incomplete penetrance and range of psychiatric presentations make *DISC1* a prime candidate for understanding how environmental factors interact with a defined genetic component to yield a variety of behavioural phenotypes.

Studies in mice have shown that DISC1 can impact on behaviour (21,22) and can also modulate reactivity to stress (21,23–28). These studies have utilised either mice with *Disc1* point mutations (27), mice carrying a naturally occurring 25 base-pair deletion in *Disc1* (29,30), or transgenic mice expressing a truncated form of human *DISC1* (24,28,31). Depending on the type of mutation or transgene used, varying phenotypes have been found, with many showing an impaired response to stress (23,24,28,31–33). Studies that have investigated the mechanism through which DISC1 and stress interact to modulate behaviour have revealed epigenetic modifications in dopaminergic neurons that originate in the ventral tegmental area (24). However, no study has examined whether *Disc1* mutation alters development of the HPA axis in a manner that impacts on stress modulation.

Expression studies in primates and mice have shown that *DISC1* orthologues are prominently expressed in the hypothalamus (34–36), a small evolutionarily conserved part of the brain that coordinates responses to stress. Analysis of *DISC1* expression in the human brain has mainly focused on the hippocampus, but expression patterns here correspond well with those in the primate and rodent hippocampus, suggesting some level of conservation (37). We previously observed strong expression of *disc1* in the ventral diencephalon of zebrafish embryos (38). We therefore reasoned that *disc1* may be required for normal hypothalamic development and functioning of the HPA axis or corresponding hypothalamic-pituitary-interrenal (HPI) axis in fish.

To address this hypothesis, we utilised two lines of zebrafish harbouring nonsense mutations in *disc1* (L115X and Y472X) and analysed baseline and stress-responsive behaviours in the adult. We investigated the developmental origin of mutant behavioural abnormalities, and show that *disc1* is essential for normal development of the early hypothalamus and HPI axis function.

## Results

### Adult *disc1* mutants exhibit anxiety-like behaviour and aberrant behavioural stress responses

**Figure 1:**
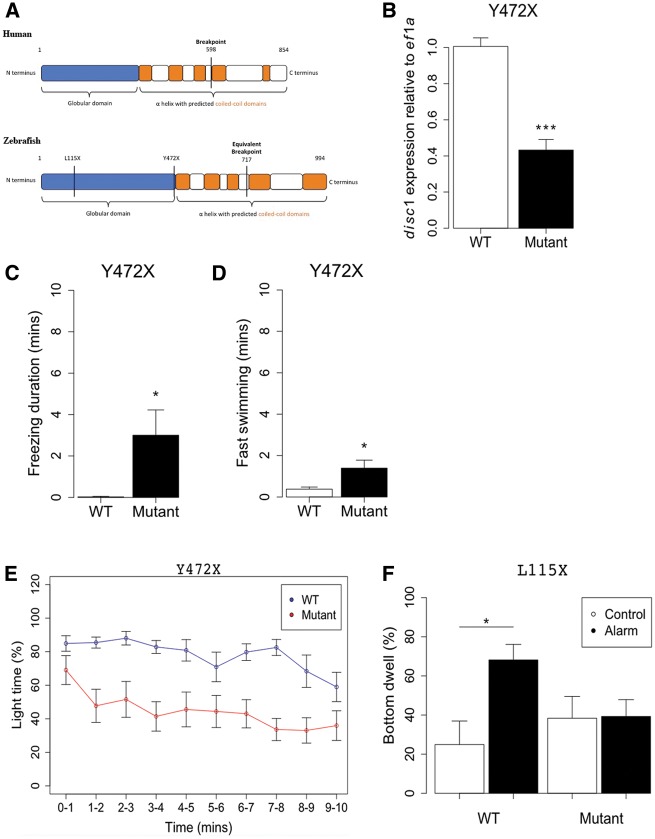
Abnormal behaviour in adult *disc1* zebrafish. (**A**) DISC1 protein schematic in human (upper) and zebrafish (lower). The predicted human DISC1 protein is 854 amino acids long, consisting of a globular *N*-terminal domain and C terminal domain with predicted coiled coil regions (19). The breakpoint observed in the original Scottish pedigree is shown at 598 amino acids. The predicted zebrafish disc1 protein is 994 amino acids long, similarly consisting of a globular N-terminal domain and C-terminal domain with predicted coiled coil regions (38). The L115X and Y472X stop codons, as well as the predicted equivalent site for the human translocation breakpoint are illustrated. (**B**) Quantitative RT-PCR for *disc1* in 2 dpf larvae shows a significant reduction in *disc1* mRNA in Y472X mutants (*t* test; *t = * 7.59, df = 9.63, *P* = <0.0001). *N = * 6 each. (**C**) Adult Y472X mutants show a significant increase in freezing in the open field test (*t* test; *t = *−2.44, df = 12.01, *P*=0.031). *N = *10–13 each. (**D**) Adult Y472X mutants show a significant increase in high speed swimming in the open field test (*t* test; *t = *−2.55, df = 13.63, *P*=0.024). *N = *10–13 each. (**E**) Adult Y472X mutants have a significantly reduced preference for the light compartment of the light-dark test (repeated measures ANOVA; Genotype, *F = *11.79, df = 1,20, *P*=0.003; time, *F = *45.29, df = 1,196, *P*≤0.0001; Genotype: time, *F = *0.41, df = 1,196, *P*=0.522). *N = *9–13 each. (**F**) Alarm substance increases bottom dwell of L115X wild types in the tank diving test (*P*=0.027), but has no effect on mutants (*P*=0.99) (two-way ANOVA; Genotype: alarm interaction, *F = *4.47, df = 1,35, *P*=0.042). *N = *9–12 each.

The L115X and Y472X mutations both introduce a premature stop codon in the *N*-terminal head domain of DISC1 (Fig. 1A). We have maintained both lines on a TL background and found that homozygous mutants are born in Mendelian ratios, hatch normally, are viable to adulthood, and fertile. Quantitative RT-PCR demonstrated that Y472X mRNA may be subject to nonsense-mediated decay (Fig. 1B). We first analysed whether, as in mice, the baseline behaviour of the adult *disc1* mutants, or their response to an acute stressor, is significantly different to wild type siblings. Adult Y472X mutants were tested for baseline behaviours and adult L115X mutants were tested for response to an established stress paradigm: exposure to alarm substance (Schreckstoff: a zebrafish skin extract that induces a profound fear response (39,40). In an open field test, adult Y472X fish showed increased freezing and increased fast swimming compared to wild type siblings (Fig. 1C–D). In a light-dark test, Y472X fish showed no preference for the light compartment, in contrast to wild type siblings (Fig. 1E). In the tank diving test, L115X mutants did not increase bottom dwell time after treatment with alarm substance, in contrast to wild type siblings (Fig. 1F). Abnormalities in baseline and stress-responsive behaviour have also been described in adult *Disc1* mouse models (24,27,28,31–33). These data show that the role of DISC1 in stress responses is evolutionarily conserved.

### Hypothalamic progenitors, including *rx3+ *progenitors, are not maintained normally in *disc1* mutant embryos

DISC1 governs neuronal progenitor proliferation (41,42), so we reasoned that early developmental abnormalities may underlie the observed adult phenotypes. Studies in mice have shown that cellular homeostasis is disrupted in the cortex of *disc1* mutants where cortical progenitors differentiate prematurely due to compromised Wnt/GSK3 signaling (41,42). Similarly, in zebrafish, zDisc1 promotes brain neurogenesis by promoting Wnt signaling (43), while a study in human induced pluripotent stem cells linked disruption of *DISC1* with altered Wnt signaling and neural progenitor cell differentiation (44). To date, however, no study has analysed progenitor cells or differentiating neurons in the hypothalamus.

**Figure 2:**
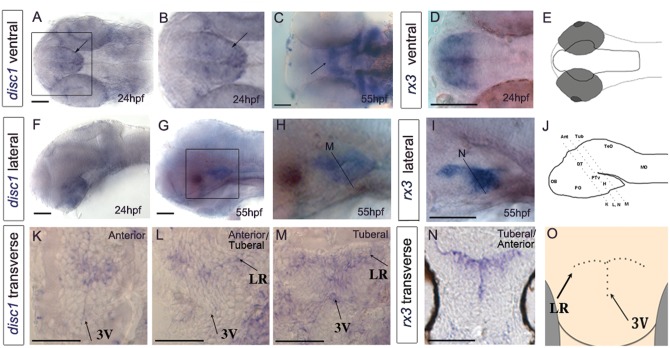
Expression of *disc1* in the larval zebrafish brain. (**A–J**) 24 hpf or 55 hpf embryos after *in situ* hybridization for *disc1* (A–C, F–H) or *rx3* (D, I) shown in ventral whole-mount view (A–D) or side view (F–I). Anterior to left. (B,H) show high power views of boxed regions in (A,G). (E,J) show schematic ventral and side views. Arrows point to expression of *disc1* in hypothalamus. Lines in side views (H–J) indicate planes of sections shown in (**K–N**). (K–N) Transverse sections taken through 55 hpf embryos after *in situ* hybridization for *disc1* (K-M) or *rx3* (N). Schematic (**O**) shows position of 3^rd^ ventricle and lateral recesses. Abbreviations: 3V, 3^rd^ ventricle; Ant, anterior; DT, dorsal thalamus; H, hypothalamus; LR, lateral recess; MO, medulla oblongata; OB, olfactory bulb; PO, preoptic region; PTv, ventral posterior tuberculum; TeO, tectum opticum; Tub, tuberal. *N = *6 each. Scale bar: 50 μm.

Analysis of *disc1* in embryonic zebrafish (24–55 h post-fertilisation (hpf)/2–3 days post-fertilisation (dpf)) revealed that expression is most prominent in the basal part of the brain, in particular the hypothalamus (Fig. 2A–C,F–H). Transverse sections through the 55 hpf hypothalamus show that *disc1* is restricted to cells around the lateral recesses and posterior tuberal 3^rd^ ventricle (Fig. 2J–M,O), neurogenic zones that harbour proliferating progenitors (45–50). Throughout this period, the expression of *disc1* is largely adjacent to that of *retinal homeobox 3* (*rx3*) a conserved paired-like homeodomain transcription factor (Fig. 2D,I,N), which, in the tuberal hypothalamus, demarcates progenitor cells that give rise to specific hypothalamic neuronal populations, including neurons of the ventromedial nucleus (VMN) and arcuate nucleus (Arc) (51,52).

**Figure 3:**
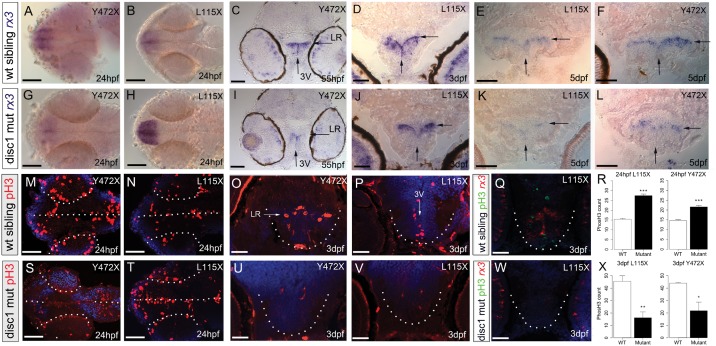
Progenitor cell alterations in the hypothalamus of *disc1* L115X and Y472X larvae. (**A–L**) *In situ* hybridization for *rx3* in wild type (**A–F**) and *disc1* mutant (**G–L**) larvae in ventral whole-mount views at 24 hpf (A,B,G,H) or representative transverse sections through posterior parts of the tuberal hypothalamus at 55 hpf (C,I), 3 dpf (D,J) and 5 dpf (E,F,K,L). Expression of *rx3* is reduced at all-time points in Y472X larvae when compared to wild types (A,C,F,G,I,L). Expression of *rx3* is elevated at 24 hpf, then reduced in L115X mutant larvae at later time points, when compared to wild types (B,D,E,H,J,K). In both mutant lines, no *rx3* is detected in the 3^rd^ ventricle and the width of *rx3* expression across the lateral recess is significantly reduced at 5 dpf (L115X, *t* test, *t = *2.80, df = 22, *P*=0.010; Y472X, *t* test, *t = *3.51, df = 22, *P*=0.002). (**M–X**) Immunohistochemical analyses for phosH3 (M–P,S–V) or dual immunohistochemical/*in situ* hybridisation analyses for phosH3 and *rx3* (Q,W), with DAPI counter-labelling (blue) in wild type (M–Q) and *disc1* mutant (S–W) larvae in ventral whole-mount views at 24 hpf (M,N,S,T) or representative transverse sections through posterior parts of the tuberal hypothalamus at 3 dpf (O–Q, U–W). Significantly more phosH3+ cells are detected at 24 hpf (L115X, *t* test, *t = *−12.51, df = 6.73, *P*≤0.0001; Y472X, *t* test, *t = *−7.73, df = 7.06, *P*=0.0001), and significantly fewer phosH3+ cells are detected at 3 dpf in mutant lines compared to wild type siblings (R,X) (L115X, *t* test, *t = *4.58, df = 7.97, *P*=0.002, Y472X, *t* test, *t = *3.17, df = 4.08, *P*=0.033). *N = *5 each. Arrows point to lateral recess (LR) or 3^rd^ ventricle (3V). In whole-mount views, dotted outlines show developing eyes and ventral midline. In transverse sections, dots outline hypothalamus. Scale bars: 50 μm.

To address whether, similar to its role in the mouse cortex, DISC1 maintains hypothalamic progenitor cells, we compared *rx3* expression in wild type and *disc1* mutant embryos and post-hatched (5 dpf) larvae. In both lines, *rx3* is altered in *disc1* mutants in comparison to wild type siblings (Fig. 3A–L; Supplementary Material, Fig. S1). In Y472X fish, *rx3* is reduced in mutant fish compared to wild type siblings at all stages examined (24 hpf-5 dpf) (Fig. 3A,C,F,G,I,L). In L115X mutant fish, *rx3* transcripts are detected at higher levels at 24 hpf than in wild type siblings (Fig. 3B and H), but from 3 dpf, L115X mutants show a similar reduction to that detected in Y472X mutants (Fig. 3D,E,J,K). Transverse sections show that *rx3* is expressed in the 3^rd^ ventricle up to 3 dpf and in the lateral recesses up to 5 dpf in wild-type fish (Fig. 3C–F,Q). By contrast, from 2 dpf, both L115X and Y472X mutant larvae show a significant reduction in *rx3* expression in the lateral recesses, and no expression can be detected in the 3^rd^ ventricle (Fig. 3I–L,W and Fig. 3 legend). This suggests that DISC1 is required to maintain progenitor cells and predicts that *disc1* mutants will show a premature decline in hypothalamic expression of phosphorylated histone H3 (phosH3), an M-phase marker whose expression correlates with proliferating progenitors in the embryonic zebrafish hypothalamus (45). At 24 hpf, a significant increase in phosH3+ cells is detected in the hypothalamus of *disc1* mutant embryos compared to wild type siblings (Fig. 3M,N,R–T). At 3 dpf, however, significantly fewer phosH3+ cells are detected in the hypothalamus of both *disc1* mutant strains compared to wild type siblings (Fig. 3O–Q,U–X). Together our analyses suggest that hypothalamic progenitors, including *rx3+ *progenitors, form, but are not maintained normally in *disc1* mutant embryos.

### Abnormal neuroendocrine differentiation and activity in *disc1* mutant embryos

We extended these experiments to determine whether the changes in progenitor proliferation lead to alterations in neuronal differentiation. Lineage-tracing studies show that *rx3+ *progenitors in the tuberal hypothalamus give rise to VMN neurons that express the nuclear receptor, *ff1b* (also termed *nr5a1a*; an orthologue of mammalian *SF1/NR5A1* (53)) and to Arc neurons that express *pro-opiomelanocortin (pomc)* (45,54). We therefore first determined if *disc1* mutant fish showed alterations in *ff1b* and *pomc*. We analysed both embryos at 2–3 dpf, a time when neuroendocrine cells are being born, and larvae at 5 dpf, a time when the neuroendocrine system begins to respond dynamically to external and internal cues (55).

**Figure 4:**
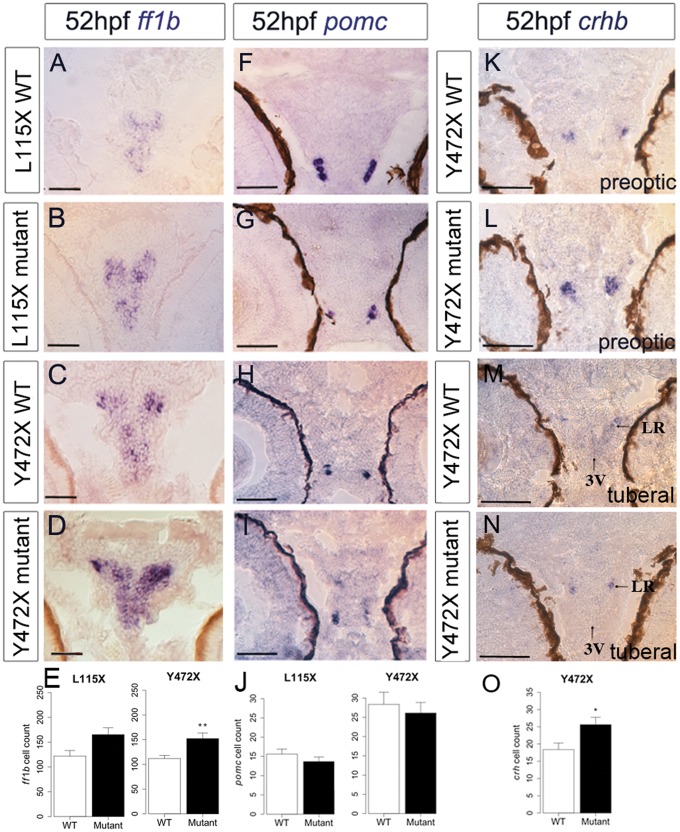
Abnormal neuronal differentiation in the hypothalamus of *disc1* L115X and Y472X embryos. (**A–D,F–I**) Transverse sections through posterior tuberal hypothalamus at 52 hpf after *in situ* hybridisation with *ff1b* (*nr5a1a*) (A–D) or *pomc* (F-I). *ff1b* is expressed more strongly in both L115X (B) and Y472X (D) mutant larvae, compared to wild types (A and C). (**E,J**) Quantitative analyses of *ff1b* and *pomc* cell number at 52hpf. *ff1b* cell count was not different in L115X embryos (*t* test, *t = *−2.45, df = 3.86, *P*=0.073, *N = *3), but was significantly increased in Y472X mutants compared to wild types (*t* test, *t = *-3.13, df = 14.06, *P*=0.007, *N = *9–10). *pomc* cell count was not significantly altered in L115X (*t* test, *t = *1.10, df = 5.56, *P*=0.318, *N = *3–5) or Y472X (*t* test, *t = *0.54, df = 25.38, *P*=0.593, *N = *13–16) embryos (J). (**K-O**) Transverse sections through preoptic (K,L) or posterior tuberal hypothalamus (M,N) at 52 hpf after *in situ* hybridisation with *crhb* in the Y472X line. Quantitative analysis (O) shows significantly more *crhb+* cells in the preoptic and tuberal hypothalamus of mutant larvae (*t* test, *t = *−2.53, df = 7.83, *P*=0.036). *N = *5 each. Abbreviations: 3V, 3^rd^ ventricle of the hypothalamus; LR, lateral recess of the hypothalamus; WT, wild type larvae; mutant, homozygous mutant larvae. Scale bar: 50 μm.

**Figure 5:**
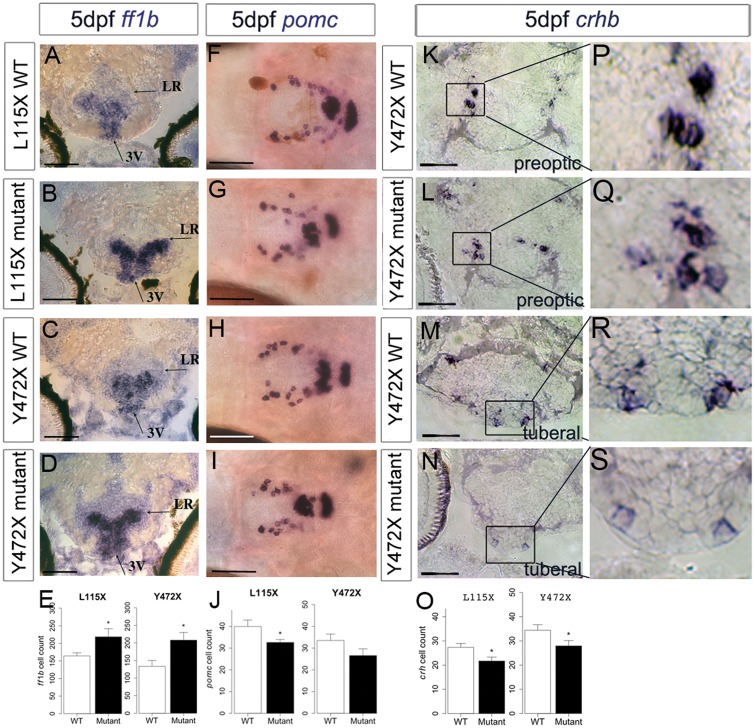
Abnormal neuronal differentiation in the hypothalamus of *disc1* L115X and Y472X larvae. (**A–E**) Transverse sections (A–D) through posterior tuberal hypothalamus at 5 dpf after *in situ* hybridisation with *ff1b*. *ff1b* is expressed more strongly in both L115X and Y472X mutant larvae, and in more cells (E) compared to wild types (L115X, *t* test, *t = *−2.20, df = 11.64, *P*=0.049, Y472X, *t* test, *t = *−2.70, df = 12.91, *P*=0.018). *N = *8–10 each. (**F–J**) Ventral whole-mount views at 5 dpf after *in situ* hybridisation with *pomc*. *pomc+* cells are disorganised in the hypothalamus of mutant larvae (G,I) compared to wild type siblings (F,H). (J) Quantitative analysis at 5 dpf shows significantly fewer hypothalamic *pomc+* cells in L115X (*t* test, *t = *2.24, df = 21.52, *P*=0.036, *N = *16) mutants compared to wild types, but no significant difference in the Y472X line (*t* test, *t = *1.63, df = 8.79, *P*=0.139, *N = *5–6). (**K–O**) Transverse sections through preoptic (K, L) or posterior tuberal hypothalamus (M,N) at 5 dpf after *in situ* hybridisation with *crhb* in the Y472X line. High power views of boxed regions show weaker expression in mutant particularly in the tuberal region (**P–S**). Quantitative analysis (O) shows significantly fewer *crhb+* cells can be detected in the preoptic and tuberal hypothalamus of both lines mutant larvae (L115X: *t* test, *t = *−2.47, df = 21.95, *P*=0.022; Y472X: *t* test, *t = *−2.08, df = 23.85, *P*=0.049. *N = *11–14 each. Abbreviations: 3V, 3^rd^ ventricle of the hypothalamus; LR, lateral recess of the hypothalamus; WT, wild type larvae; mutant, homozygous mutant larvae. Scale bar: 50 μm.

In wild type embryos, *ff1b* is first detected in the developing hypothalamus at 24 hpf (56). The role of *ff1b* in the zebrafish hypothalamus has not been determined, but in mice, hypothalamic *Sf1* governs anxiety behaviours (57). In both embryos and larvae, expression of *ff1b* was significantly more pronounced in the hypothalamus of L115X and Y472X mutants compared to wild type siblings (Figs. 4A–D, 5A–D; Supplementary Material, Fig. S2A–D) and was detected in greater numbers of cells (Figs. 4E and 5E). Thus, the failure to maintain *rx3+ *progenitors appears to correlate with an enhanced differentiation of hypothalamic *ff1b+ *cells. *ff1b* is also expressed in steroidogenic cells of the interrenal gland, and is essential for proper development of this tissue (58). We observed normal expression of *ff1b* in this region of *disc1* mutants (Supplementary Material, Fig. S2M–P).

The precursor peptide, proopiomelanocortin (pomc) defines Arc-like neurons in the hypothalamus. In wild type zebrafish, hypothalamic *pomc+ *neurons are detected from 32 hpf, i.e. some hours after *ff1b+ *cells (59). Analysis over 2–3 dpf revealed no significant difference in number of hypothalamic *pomc+ *cells at each time (Fig. 4F–J;Supplementary Material, Fig. S2E–H, K), but at 5 dpf, significantly fewer *pomc+ *neurons were detected in L115X mutants (Fig. 5J). In both L115X and Y472X mutants, *pomc+ *neurons appeared disorganised, and were not detected in the characteristic horseshoe pattern found in the wild type animals (Fig. 5F–I).

We next examined whether *disc1* affects *corticotropin releasing hormone (crhb)-*expressing neurons, a population that is specified in an *rx3*-independent manner (60), but that plays an instrumental role in the stress response. Analysis of *crhb* in Y472X mutants revealed embryonic expression in both the preoptic and tuberal hypothalamus, as shown in previous studies (61,62). At 2–3 dpf, increased numbers of *crhb*+ neurons were detected in mutant embryos, compared to wild types, in both domains (Fig. 4K–O, Supplementary Material, Fig. 2I–J, L). By contrast, at 5 dpf, a time when neuroendocrine-responsive transcriptional programmes adapt dynamically to the supply and demand for neuropeptides, significantly fewer *crhb+ *neurons could be detected in mutant larvae (Fig. 5O), and where expression was detected, it was weak, relative to that in wild type siblings, particularly in tuberal regions (Fig. 5K–S).

Taken together, our results suggest that DISC1 is required to determine appropriate numbers of hypothalamic progenitors, including *rx3+ *progenitors, and appropriate numbers/position of differentiated neurons, including *ff1b+, pomc+ *and *crh+ *neurons.

### *disc1* mutant larvae show impaired behavioural responses to stress

**Figure 6:**
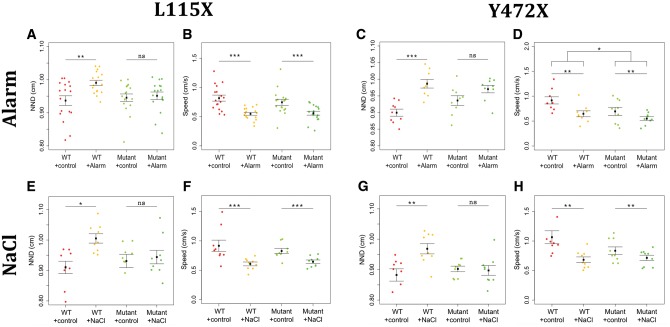
Shoaling behaviour of 5 dpf *disc1* L115X and Y472X larvae is modulated by chemical stressors. (**A–D**) Effect of alarm substance on *disc1* L115X and Y472X larval behaviour. Exposure to alarm substance caused an increase in NND of L115X (*P*= 0.009) and Y472X (*P*=0.0001) wild type larvae but not mutants (L115X, *P*=0.985; Y472X, *P*=0.228) (A, C). Exposure resulted in a decrease in swim speed of both wild type and mutant L115X (two-way ANOVA, *F = * 29.63, df = 1, 64, *P* = <0.0001, *N = *17) and Y472X (two-way ANOVA, *F = *10.06, df = 1,32, *P*=0.003, *N = *9) larvae (B, D). Y472X mutants swam slower than wild types (two-way ANOVA, *F = *5.98, df = 1,32, *P*=0.020, *N = *9) (D). (**E–H**) Effect of osmotic shock on *disc1* L115X and Y472X larval behaviour. Exposure to sodium chloride caused an increase in NND of L115X (*P*=0.010) and Y472X (*P*=0.003) wild type larvae but not mutants (L115X, *P*=0.968; Y472X, *P*=0.996) (E,G). Exposure resulted in a decrease in swim speed of both wild type and mutant L115X (two-way ANOVA, *F = *18.49, df = 1, 64, *P*=0.0002, *N = *9) and Y472X larvae (two-way ANOVA, *F = *12.32, df = 1,32, *P*=0.001, *N = *9) (F,H).

*ff1b* orthologues are implicated in anxiety-like behaviours in mice (57) and depression and anxiety in humans (63), whilst *crh* orthologues are implicated in anxiety-like behaviours in mice (15,64) and primates (65), and in depression in humans (66). The altered differentiation (and, potentially activity) of *ff1b+ *and *crhb+ *neurons, together with the altered stress reactivity in *disc1* adult mutants, led us to ask whether altered behavioural responses to stress in *disc1* mutants are established early in development. We analysed each mutant strain for behavioural responses to two established stress paradigms, osmotic stress (67) and exposure to alarm substance (39,40). At 3 dpf embryos already exhibit increased cortisol levels in response to severe stress (61), while at 5 dpf, larvae display anxiety-related behaviours such as thigmotaxis and dark avoidance (68). Acute exposure to either alarm substance or sodium chloride at 5 dpf resulted in a significant increase in the nearest neighbour distance (NND, a measure of shoal cohesion) in wild type larvae, but did not affect NND in either L115X or Y472X mutants (Fig. 6A,C,E,G). By contrast, exposure to the stressors caused a significant reduction in swimming speed of both wild type and mutant *disc1* larvae (Fig. 6B,D,F,H). L115X mutant swimming speed was not different to that of wild types, but Y472X mutants swam more slowly than wild types (Fig. 6D). To summarise, whilst both wild type and mutant larvae swam more slowly when under stress, only wild type larvae modulated shoal cohesion in response to stressors.

The failure to modulate shoaling behaviour in response to stress by *disc1* mutants could be due to abnormalities in lateral line development or the visual system, either of which could impact on shoaling behaviour (69,70). However, when exposed to a short pulse of darkness, both wild type and mutant larvae exhibit a startle response (Supplementary Material, Fig. S3A). Furthermore, analysis of FM1-43, a marker of neuromasts of the lateral line, likewise showed similar numbers in both wild type and mutant larvae (Supplementary Material, Fig. S3B). Thus, gross defects in two sensory systems - the visual system and the lateral line – do not appear to account for the impaired shoaling behaviour.

### Environmental stress fails to trigger the HPI axis in *disc1* mutant larvae

**Figure 7:**
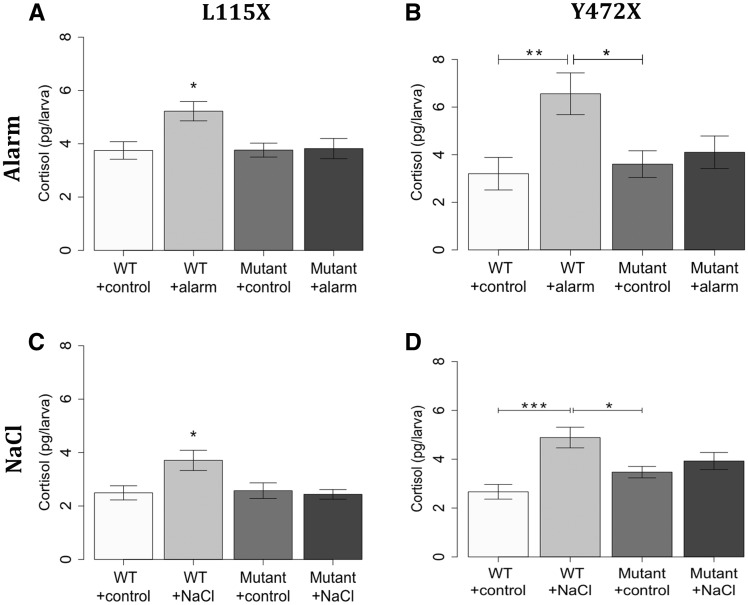
Effect of exposure to chemical stressors on whole body cortisol levels of 5 dpf *disc1* L115X and Y472X zebrafish larvae. **(A,B)** Exposure to alarm substance increased cortisol level in wild type L115X (A) (*P*=0.024, *N = *15–17) and Y472X (B) (*P*=0.008, *N = *8–11) larvae, but not mutants (*P*=0.999 and 0.960 respectively). *Different to all other groups at *P*<0.05. **(C,D)** Exposure to sodium chloride increased cortisol level in wild type L115X (C) (*P*=0.026, *N = *9) and Y472X (D) (*P*=0.0003, *N = *8) larvae, but not mutants (*P*=0.987 and 0.769, respectively). *Different to all other groups at *P*<0.05.

The altered behavioural reactivity to stress in the *disc1* mutant larvae led us to postulate that endocrine responses might be impaired. We therefore measured cortisol levels with and without stress exposure. As anticipated (67), exposure to either alarm substance or sodium chloride led to a significant increase in whole body cortisol levels in wild type larvae. By contrast, exposure to either alarm substance or sodium chloride had no significant effect on whole body cortisol levels of either *disc1* L115X mutant larvae (Fig. 7A and C) or Y472X mutant larvae (Fig. 7B and D). No significant differences were observed in baseline cortisol levels between wild type and *disc1* mutant larvae. Together, these results show that mutations in *disc1* prevent the normal functioning of the HPI axis, in particular, the cortisol-mediated stress response.

## Discussion

Humans that carry a mutation in *DISC1* present with a variety of psychiatric conditions, including depression, schizophrenia and bipolar disorder (19). Mouse *disc1* mutant models similarly exhibit behavioural abnormalities (21,22,27). Our studies reveal behavioural abnormalities in adult *disc1* mutant zebrafish that include freezing in the open field and a reduced preference for the light compartment in the light-dark test. What might these behaviours represent, and are they relevant to understanding the pathobiology of psychiatric disorders? Freezing behaviour is commonly observed in zebrafish after exposure to stressors such as alarm substance and is typically accompanied by darting and erratic movements (39). This combination of behaviours has been considered an anxiety-like behaviour in zebrafish (71). Moreover, freezing in the open field test is a characteristic behaviour of the zebrafish glucocorticoid receptor mutant (16), where, unaccompanied by darting, it is considered to represent a depressive-like behaviour. Since, in the open field, *disc1* mutant zebrafish exhibit increased freezing, and high speed darting in between freezing periods, we believe that behaviour in this test indicates increased anxiety. Further support for this conclusion comes through analysis of the response to the light-dark test. Wild type zebrafish strongly prefer the light compartment, a behaviour that is likely to be advantageous, in that the light allows for easier detection of food, mates and predators (68); the light compartment is also more familiar, due to its similarity to the home tank. A reduction or reversal of preference for the light compartment has previously been detected in zebrafish that have been stressed (72,73). Further, preference for the light compartment is exacerbated by exposure to anxiolytic drugs (74). Together, the decreased preference for the light compartment shown by the *disc1* Y472X mutants supports the notion that they exhibit increased anxiety-like behaviour.

DISC1 is known to interact via its *N*-terminal globular domain with PDE4B, mutation of which has previously been implicated in neurodevelopmental disorders such as schizophrenia (75). A recent study revealed that treatment of zebrafish larvae with the PDE4-specific small molecule inhibitor Rolipram elicited robust, anxiety-like and hyperactive behaviours (76). Taken together with our findings, these results suggest that Disc1-PDE4 protein complexes may perform anxiolytic functions in the zebrafish brain, disruption of which could increase the risk of developing a psychiatric disorder. Interestingly, analysis of *pde4d* homozygous mutant zebrafish indicates that *pde4d* performs an anxiogenic function in wild-type fish (76). Thus, it seems likely that Disc1 protein might interact with a PDE4 orthologue other than Pde4d, such as Pde4b, to limit anxiogenic behaviour in zebrafish. Future biochemical studies of the formation of Disc1-Pde4 complexes in zebrafish may help to address this question.

The diversity of psychiatric conditions presented in the *DISC1* pedigree, suggests that DISC1 function, or downstream effectors, might be modulated by environmental signals. Indeed, mouse models of *DISC1* display abnormal stress responses (21,23–28). We found that acute exposure to alarm substance increased bottom dwell time in wild type zebrafish, as previously described (39,77), but had no effect on *disc1* mutants. Increased bottom dwell is generally considered to be indicative of increased anxiety, and, indeed, this measure is sensitive to anxiolytic drugs (74). By this logic, a failure to increase bottom dwell duration by *disc1* L115X mutants could be considered as reduced anxiety, but this explanation seems at odds with the previously discussed anxiety-like behaviours that were detected in the Y472X mutants in the open field and light-dark tests. We therefore hypothesise that *disc1* mutants may have an impairment in their detection or processing of stressful stimuli.

The observed anxiety-like behaviours and failure to activate appropriate stress responses in the adult *disc1* mutant zebrafish led us to question whether *disc1* functions in the hypothalamus, the key regulator of the stress axis, and whether the observed behavioural defects have a developmental origin. We found that *disc1* is expressed in the developing hypothalamus in proliferating progenitor cells that line the posterior part of the 3^rd^ ventricle and lateral recesses (45,51). Studies in mice have shown that in *Disc1* mutants, progenitor cells in the cortex exit the cell cycle, and differentiate, prematurely (41). Our studies suggest that *disc1* may play a similar role in the embryonic zebrafish hypothalamus: proliferation is initially enhanced, then prematurely reduced, in mutant embryos compared to wild type siblings. Likewise, *in situ* hybridisation reveals reduced expression of *rx3*, a marker of anterior/tuberal hypothalamic progenitors, in *disc1* mutants. Previous studies have shown that *rx3* is required for specification of *ff1b*-positive (the zebrafish homologue of SF1/NR5A1) and *pomc-*positive neurons (51), and we find that alterations in *rx3+ *progenitor cells in *disc1* mutants have downstream effects on each of these neuronal classes: *ff1b*-positive cells, which differentiate early, increase in number, while later-born *pomc*-positive neurons are disorganised, and, in the L115X line, reduced in number. In mice and zebrafish, a complete loss of *Rax*/*rx3* function leads to loss of *Sf1/ff1b*-positive cells (45,51), and so at first glance it is surprising that the reduced *rx3* expression observed in *disc1* mutants correlates with enhanced *ff1b* expression. However, studies in fish show that *rx3* must be downregulated in progenitor cells for them to realize their differentiation programme (45). This further supports the idea that *disc1* mutants show a premature differentiation of *rx3-*positive progenitor cells, and suggests that *ff1b-*positive neurons (which normally differentiate early) are preferentially increased in number in *disc1* mutant fish. In summary, our studies suggest that in zebrafish, *disc1* is required for proliferation of *rx3*-positive progenitors, with loss of *disc1* function leading to premature differentiation and early excessive production of *ff1b*-positive neurons.

Several studies have linked *SF1*/*NR5A1* to anxiety. Central nervous system-specific knockout of *Sf1* in mice leads to increased anxiety-like behaviours (57), whilst more recently, down-regulation of glutamatergic output from the VMN, which harbours *Sf1*-positive neurons, was shown to have an anxiety-reducing effect (78). *NR5A1* mutations have also been linked with anxiety and depression in humans (63). Therefore, increased *ff1b/nr5a1a* expression in *disc1* mutants might indeed be expected to have behavioural consequences. Further studies are needed to determine whether upregulated expression of *ff1b* in the hypothalamus plays a direct role in the impaired stress response in *disc1* mutant larvae.

An additional possibility is that the impaired stress response that we detect in *disc1* mutant fish reflects a broader altered hypothalamic development. Expression of *disc1* is not restricted to *rx3*-positive progenitor cells, suggesting that other neuronal subsets, whose differentiation occurs independently of rx3, may develop abnormally in the mutant fish. In support of this idea, we detected a significant increase in the number of *crh-positive* neurons in *disc1* mutant embryos, followed by a significant reduction in *crhb* in *disc1* mutant larvae. These results are consistent with a model in which inappropriately high crh levels in embryos lead to unspent neuropeptide cargo, that feeds back to reduce transcription in larval neuropeptidergic cells just as they become functionally required (55). Mouse models suggest a vital role for appropriate Crh levels in normal stress regulation. Under stress, *Crh* knockout mice have impaired production of corticosterone, suggesting that Crh is essential for the normal adrenal response to stress (79). These studies raise the possibility, therefore, that alterations in crh in *disc1* mutant larvae could play a direct role in aberrant stress responses.

Our studies reveal that *disc1* mutant larvae display altered behavioural and endocrine responsiveness to acute stress. Zebrafish are a shoaling fish species, in which individuals aggregate, often with a common direction. Shoaling behaviour has been reported in larvae, soon after hatching (80). When exposed to alarm substance or NaCl, wild type larvae reduce shoal cohesion, likely a stimulus avoidance response, which, in the case of alarm exposure, would confuse the predator (81). In contrast, mutant larvae appear to have a defect in this behaviour: when stressed, *disc1* mutant larvae fail to modulate shoal cohesion. Defects in brain and muscle development were previously reported in L115X mutant zebrafish (43). We did not observe morphological abnormalities in L115X homozygotes and their baseline swimming speed was normal (Fig. 6). These differences in phenotype may reflect differences in the genetic backgrounds on which the mutants were maintained (AB vs. TL). Furthermore, our studies indicate normal locomotor behaviour in response to light stimulus, and normal numbers of lateral line neuromasts, arguing against the possibility that the failure to modulate shoal cohesion is underlain by a defect in vision or mechanoreception. Instead, it raises the possibility that failure to modulate shoal cohesion indicates a reduced social interaction, as has been demonstrated in a DISC1 mouse model (28). Such changes are likely to impact negatively on the fitness of an animal, and in humans may manifest in psychiatric disease.

In support of this idea, both *disc1* mutants failed to upregulate cortisol when stressed. Under basal conditions, cortisol levels were not significantly different between mutant and wild type larvae, indicating that the differences observed in response to stress represent a failure to activate the HPI axis, rather than an inability to synthesise cortisol. Reduced corticosterone release in response to an acute stressor was seen in DISC1 transgenic mice after maternal prenatal immune activation, compared with control mice (28). In contrast, a different transgenic DISC1 mouse model showed hyper-responsivity to stress (24). In this gene-environment interaction model, isolation stress did not lead to increased corticosterone levels in wild type mice, but did lead to increased corticosterone levels in DISC1 transgenics. While these mouse models vary in the promoters used to drive DISC1 expression and the stress paradigms used, both studies, as well as our studies with zebrafish, support the conclusion that DISC1 interacts with the HPA axis, and that aberrant DISC1 function results in altered responses to stress.

In conclusion, our data suggest that *disc1* is essential for enabling normal stress responses, including stress-sensitive social behaviour, which is likely mediated, at least in part, by altered hypothalamic development. Future studies aimed at evaluating stress responses in adults may provide insight into the dynamic action of *disc1* in the HPA axis throughout the life-course. Our studies demonstrate that the *disc1* mutant zebrafish is a valuable system in which to study gene-environment interactions and the molecular pathways underlying psychiatric disorders.

## Materials and Methods

### Zebrafish husbandry

Adult zebrafish were maintained with a 14 h light/10 h dark cycle at 28ºC according to standard protocols and were mated in groups using spawning tanks or paired using individual cross tanks (82). Both lines of *disc1* mutant zebrafish were identified in an ENU mutagenesis-based screening programme and have been reported elsewhere (43). We obtained them as an F3 outcross from Dr Cecilia Moens (Fred Hutchison Cancer Research Center, Seattle, WA) and all fish used in this study were outcrossed with the TL strain to F7/F8 generations prior to in-crossing. We refer to the *disc1^fh291^* allele as L115X and the *disc1^fh292^* allele as Y472X throughout. Larvae were obtained from in-crosses of *disc1* wild type and in-crosses of *disc1* homozygous mutant adult siblings. For behavioural analysis, 21 larvae were maintained per petri dish in E3 medium at 28.5 °C and staged according to Kimmel’s guide (83). All procedures involving experimental animals were performed in compliance with local and national animal welfare laws, guidelines and policies.

### Quantitative RT-PCR

Pools of 40 whole larvae were snap frozen at 2.5 dpf. RNA extraction, cDNA synthesis and qRT-PCR were carried out as previously described in Boyd *et al.* 2015 (84).

### Behavioural analysis

Zebrafish were transferred to the behavioural analysis room and left to acclimatise for 1 h prior to analysis. Alarm substance, the osmotic stressor (250 mM NaCl) or a control solution (water) were pipetted into the centre of the fish container and movements were tracked for 10 min. Alarm substance was extracted according to the method of Schirmer and colleagues (85) and used at a concentration of 200 μl/l swimming medium. Zebralab software (Viewpoint, France) was used to track the movement of larval and adult zebrafish and provide quantitative measures of their behaviour.

#### Adult behaviour

The open field and dark-light test tank was 25 x 15 x 15 cm and filled to 4.1 L, whilst the trapezoid tank diving test tank was 23.5 x 6.2 x 13.5 cm and filled maximally. The open field and tank diving tanks were bare, whilst the light-dark tank was half covered in a black opaque material on all sides. Fish were acclimated to the open field tank for 1 h prior to filming, whilst filming in the light-dark and tank diving tests began immediately after the fish was transferred to the tank. Light time is the percentage of time each fish spends in the light compartment, whilst bottom dwell is the percentage of time a fish spends in the lower half of the tank diving test tank.

#### Larval behaviour

Nearest-neighbour distance (NND) was quantified based on the formula provided by Miller & Gerlai (86). Briefly, NND is the mean distance of each larva to the nearest larva and is a measure of shoal cohesion, with lower values indicating greater cohesion. Swimming speed, the mean velocity of all larvae, was also measured in order to determine the motor response of larvae to the stressor.

## Whole-body cortisol assay

Immediately following behavioural analysis, the larvae in a single petri dish were pooled in to a single tube and snap frozen in liquid nitrogen. Whole-body cortisol was extracted and measured according to the ELISA-based method developed by Yeh *et al.* (67). Cortisol standards were analysed in triplicate.

## Whole-mount *in situ* hybridisation

Whole-mount *in situ* hybridisation was performed according to standard protocols (87). The following riboprobes that mark hypothalamic regions were used: *rx3* (88); *disc1* (38); *ff1b/nr5a1a* (provided by V. Laudet, Pierre and Marie Curie University, Paris, France), *pomc* (45), *crhb* (provided by W. Norton, Leicester University, UK). For analysis of *disc1*, eyes were removed after fixation. After staining, larvae were re-fixed then transferred to 30% sucrose for cryosectioning. Specimens were mounted in OCT and 12 μm thick transverse sections through the entire forebrain, were serially collected. Sense probes were routinely used as controls.

## Section analysis

Section position was determined on the basis of serial number, and relative to defined morphological landmarks (optic commissure, optic vesicles, lateral ventricle, posterior hypothalamus and adenohypophysis). This enabled accurate matching of sections from wild type and mutant siblings. Note that in some cases, optic vesicles were displaced upon cryosectioning. Width across the lateral recess, in which *rx3* was expressed, was quantified manually by measuring the distance from the end point of the lateral recess, to the point where it joins the 3^rd^ ventricle, for each section of the mid hypothalamus. The number of labeled cells in the hypothalamus was manually counted in cryosections after labeling via whole-mount *in situ* hybridization. The total number of labeled cells for each individual was counted using all hypothalamic sections, identified using morphological landmarks as described above.

## Immunohistochemistry

Fixed embryos or sections were labeled using anti-phosH3 (06-570, Millipore) at 1:1000, as described by Muthu *et al.* (45), and mounted in VectaShield.

## Image acquisition

Images of whole-mount and sectioned zebrafish were acquired using an Olympus BX60 microscope using Q Capture Pro 7.0 (QImaging). Images were processed using Adobe Photoshop CC 2014.

## Statistical analysis

Statistical analysis and graphics were created in ‘R’ Version 3.3.0 (89). Data were tested for equal variance and normality prior to analysis. Statistical significance was tested using unpaired *t* tests, for comparisons between two samples, whilst samples classified by two or more different types of treatment were analysed by Analysis of Variance (ANOVA) and *post hoc* analysis via Tukey’s test. For quantitative RT-PCR, ΔCT values for disc1 were calculated relative to ef1a for each sample. Fold change was calculated for each sample relative to the mean of the control group (wild type). Absorbance readings of cortisol standards were used to create a standard curve. Cortisol concentrations of experimental samples were determined by interpolation using a 4-parameter non-linear regression curve fit. In all cases, standard error of the mean is reported and *P < 0.05, **P < 0.01, ***P < 0.001.

## Supplementary Material

Supplementary Material is available at *HMG* online.

## Supplementary Material

Supplementary Data
