# Supplementary material for: Disrupted-in-Schizophrenia-1 is essential for normal hypothalamic-pituitary-interrenal (HPI) axis function
Source: Hum Mol Genet. 2017 Mar 1;26(11):1992–2005. doi: 10.1093/hmg/ddx076 (PMC5437527; doi:10.1093/hmg/ddx076)
Supplement: Supplementary Data [file ddx076_Supp.doc]

**SUPPLEMENTARY MATERIAL**


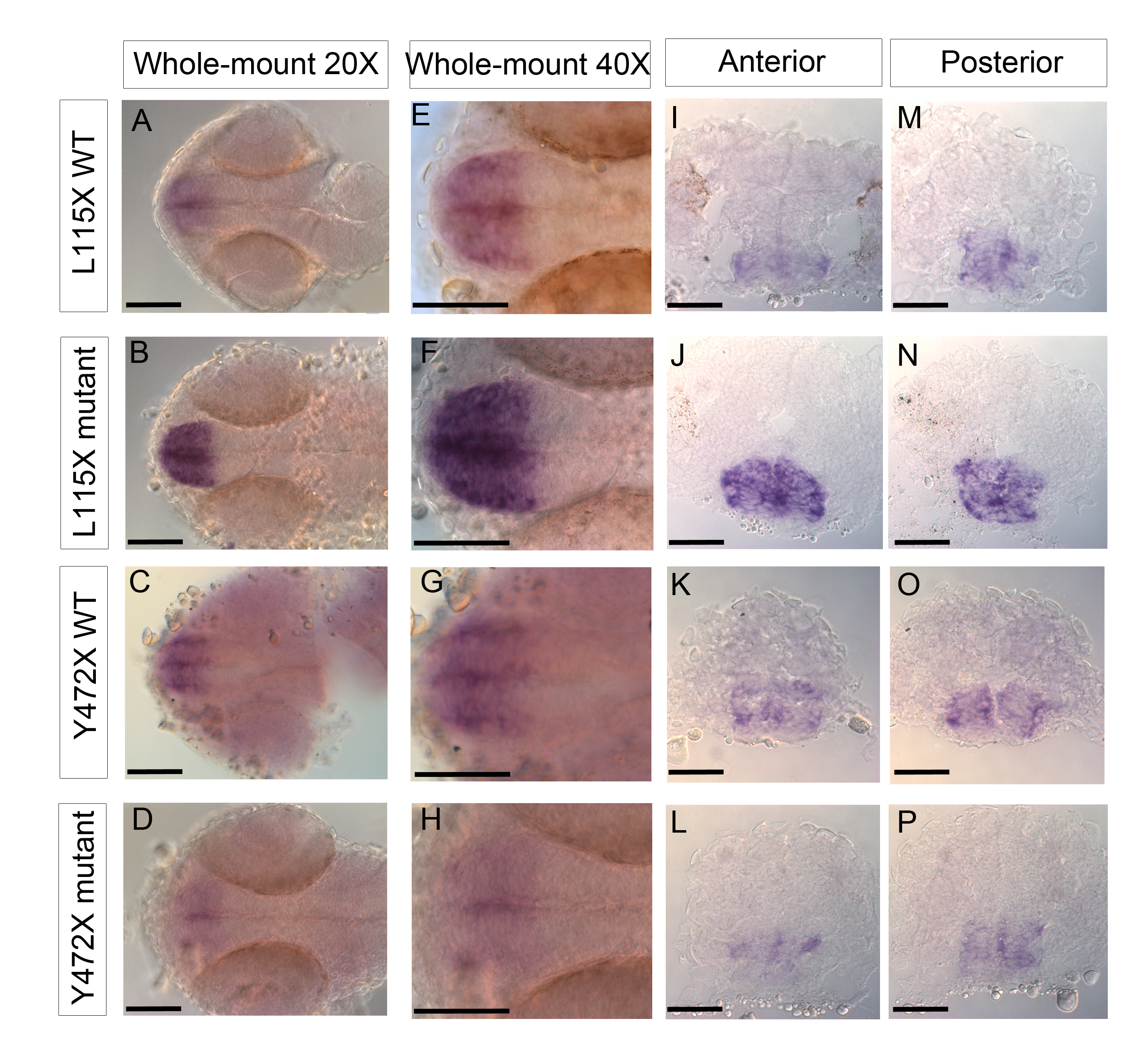


**Supplementary Figure 1. Expression of *rx3* by *in situ* hybridization in 24 hpf *disc1* embryos.** (A-H) Ventral whole-mount view of *rx3* in the developing hypothalamus, showing increased expression in L115X mutants (B,F) compared to wild types (A,E) and reduced expression in Y472X mutants (D,H) compared with wild type embryos (C,G). (A-D) are 20X magnification, (E-H) are 40X magnification. Anterior left. (I-P) Representative transverse sections through the anterior (I-L) and posterior (M-P) regions of the developing hypothalamus in L115X (I, J, M, N) and Y472X (K, L, O, P) embryos. Scale bars: 50 μm.

**
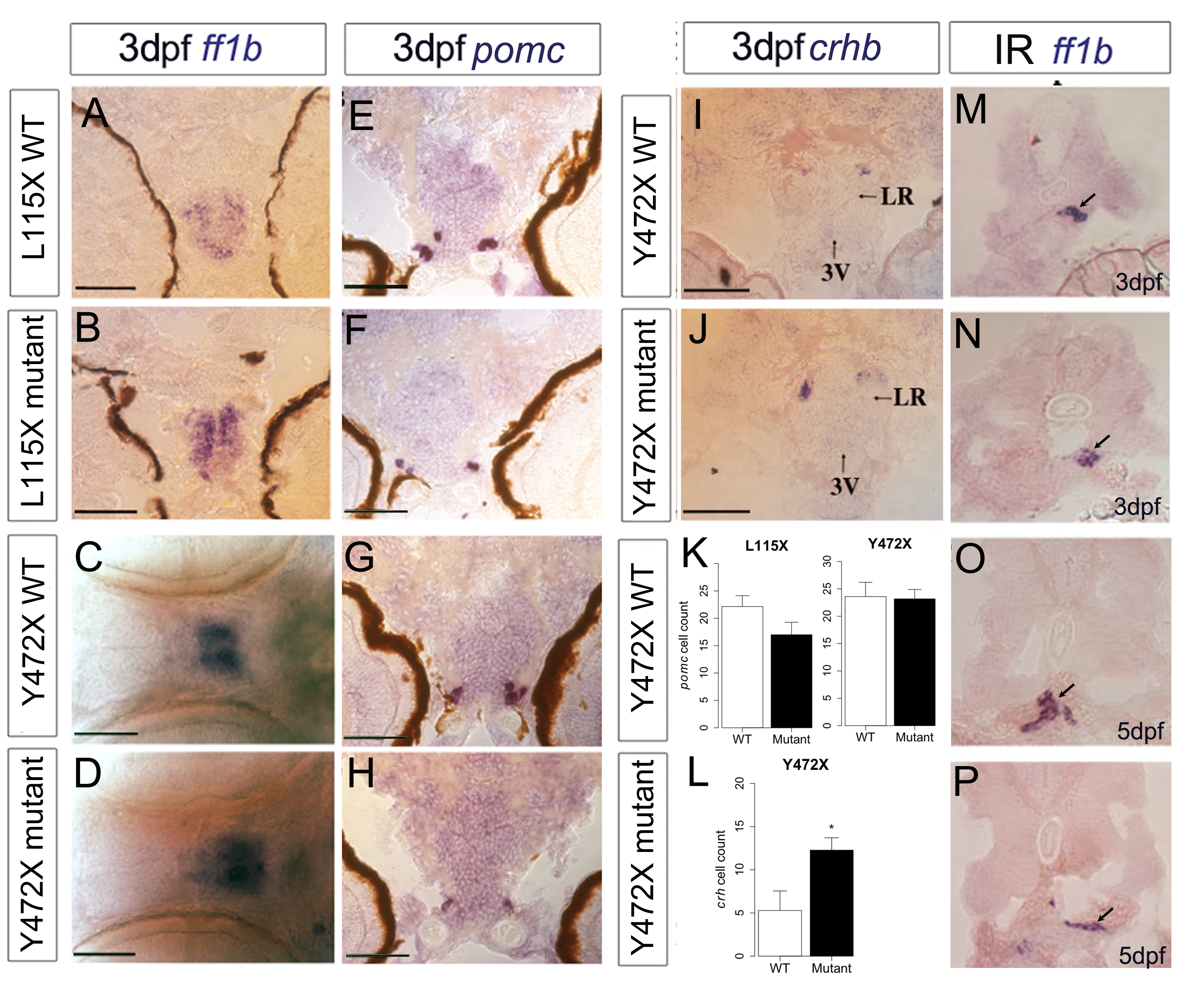
**

**Supplementary Figure 2. Expression of markers of hypothalamic neurons and steroidogenic cells in *disc1* larvae.** (A-D) Expression of *ff1b* in the hypothalamus of 3 dpf *disc1* larvae shown in transverse section in the L115X line (A, B) and ventral whole-mount view in the Y472X line (C, D, anterior left). (E-H, K) Expression of *pomc* in the hypothalamus of 3 dpf L115X (E-F) and Y472X (G-H) larvae in transverse sections. (K) Quantitative analysis of *pomc* cell count in 3 dpf *disc1* larvae showed no significant difference (L115X, t test, t=-1.71, df=21.54, p=0.103; Y472X, t test, t=0.13, df=19.08, p=0.895) N=11-14. (I-J, L) Expression of *crhb* in the preoptic and anterior hypothalamus of 3 dpf Y472X larvae. Quantitative analysis revealed significantly more *crhb* cells in Y472X mutants (t test, t=-2.61, df=10.70, p=0.025). N=7-11 each. (M-P) Expression of *ff1b* in the interrenal tissue (arrows) of *disc1* Y472X larvae shown in transverse sections at 3 dpf (M, N) and 5 dpf (O, P). N = 6 each. Scale bars: 50 μm. 3V, 3^rd^ ventricle, LR, lateral recess, IR, interrenal tissue.


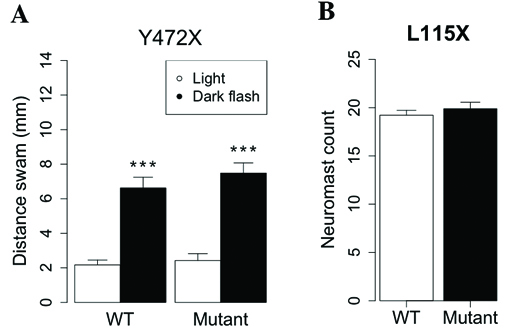


**Supplementary Figure 3. Response to a visual stimulus and lateral line analysis in *disc1* mutant larvae.** (A) Y472X wild type and mutant larvae exhibit a motor startle response to a dark flash (two-way ANOVA; Genotype F=1.13, df=1,70, p=0.293, Stimulus F=106.66, df=1,70, p=<0.0001, Genotype: stimulus interaction F=0.43, df=1,70, p=0.513). N=36 each. (B) Number of neuromasts in the lateral line of 8 dpf L115X larvae is not significantly different between wild types and mutants (t test, t=-0.80, df=29.38, p=0.428). N= 16 each.
